# Nesting and Hibernation Host Preference of Bamboo Carpenter Bee, *Xylocopa* (*Biluna*) *tranquebarorum tranquebarorum*, and Arthropods Co-Habiting and Re-Using the Bee Nest

**DOI:** 10.3390/insects16080807

**Published:** 2025-08-04

**Authors:** Natsumi Kanzaki, Keito Kobayashi, Keiko Hamaguchi, Yuta Fujimori

**Affiliations:** 1 Kansai Research Center, Forestry and Forest Products Research Institute (FFPRI), 68 Nagaikyutaroh, Momoyama, Fushimi, Kyoto 612-0855, Japan; kobayashi_keito380@ffpri.go.jp (K.K.); hamaguchi_keiko350@ffpri.go.jp (K.H.); 2 School of Agriculture, Meiji University, 1-1-1 Higashi-Mita, Tama, Kawasaki 214-8571, Japan; yutainsecta@gmail.com

**Keywords:** co-habitant, ecological impact, invasive species, natural history, nesting

## Abstract

The bamboo carpenter bee, *Xylocopa* (*Biluna*) *tranquebarorum tranquebarorum*, is native to continental China and Taiwan, and the species invaded Japan around 2006. The bee utilizes bamboo culm for its nesting and hibernation, thereby causing structural damage to bamboo fencing and sting injuries to humans. Serious economic and ecological impacts were not expected in the early stage of its invasion. However, its distribution is rapidly expanding in Japan, and thus, its potential impacts need to be evaluated. Since the basic biology of the bee has not been examined in detail, even in its natural range, we examined the basic biology of *X. t. tranquebarorum* in its invasive range by evaluating its nesting preference and hibernation in several bamboo species collections in Kyoto, Japan. The field survey revealed that the bee prefers dead bamboo internodes with approximately16–28 mm of external diameter, which is well-congruent with previous studies, and does not have strict preference concerning the bamboo species, though the bee prefers *Bambusa multiplex* and *Phyllostachys* spp. in its native range. The hibernating bees in the culm sometimes share their nests with other invertebrates, including *Anterhynchium gibbifrons*, *Dinoderus japonicus*, *Crematogaster matsumurai*, unidentified spiders, shield bugs, and lepidopteran larvae. Within these co-habitants, the former two possibly negatively affect nesting and hibernation of the bees.

## Simple Summary

The nesting and hibernation behaviors of an invasive bamboo carpenter bee, *Xylocopa* (*Biluna*) *tranquebarorum tranquebarorum*, were surveyed in Kyoto, Japan. Although the bee prefers several bamboo species, e.g., *Bambusa multiplex* and *Phyllostachys* spp. in its native range, the survey revealed that it does not have a strict preference for host bamboo species in its invasive range (Japan) but prefers a dead bamboo internode with approximately 16–28 mm of diameter. The hibernating bees sometimes share their nests with several different species of arthropods. Within these arthropods, a wasp, *Anterhynchium gibbifrons* and a powder-post beetle *Dinoderus japonicus* may negatively affect the bees through competition for a hibernating (overwintering) place (i.e., *A. gibbifrons*), or by stuffing the internode with its frass (i.e., *D. japonicus*).

## 1. Introduction

**Figure 1:**
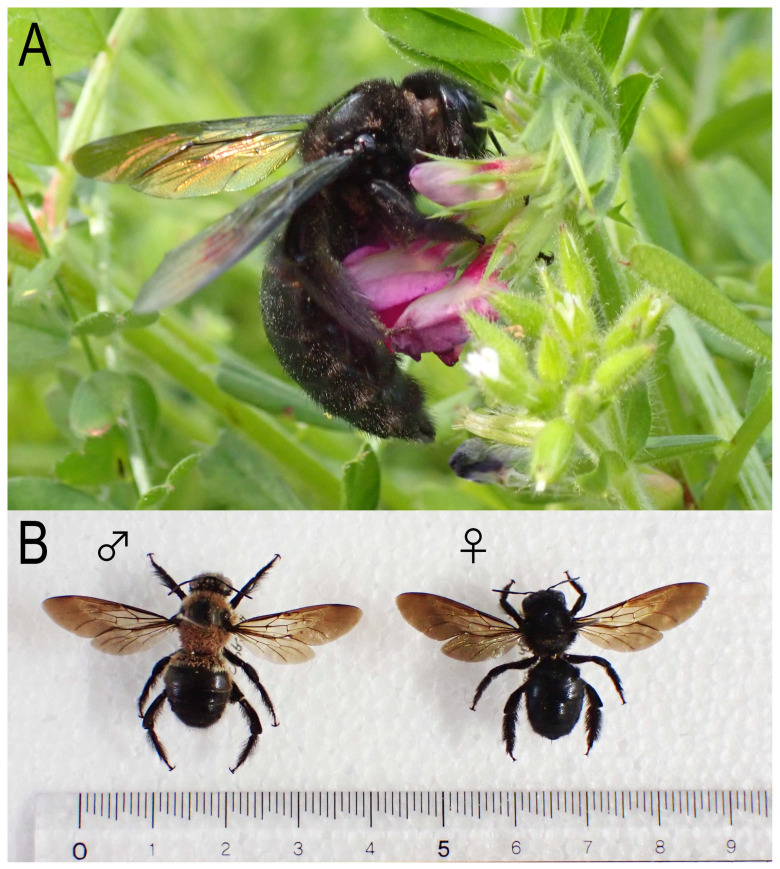
Adults of *Xylocopa* (*Biluna*) *tranquebarorum tranquebarorum*. (**A**) Foraging female; (**B**) male and female specimens. Scale in B = mm/cm.

**Figure 2:**
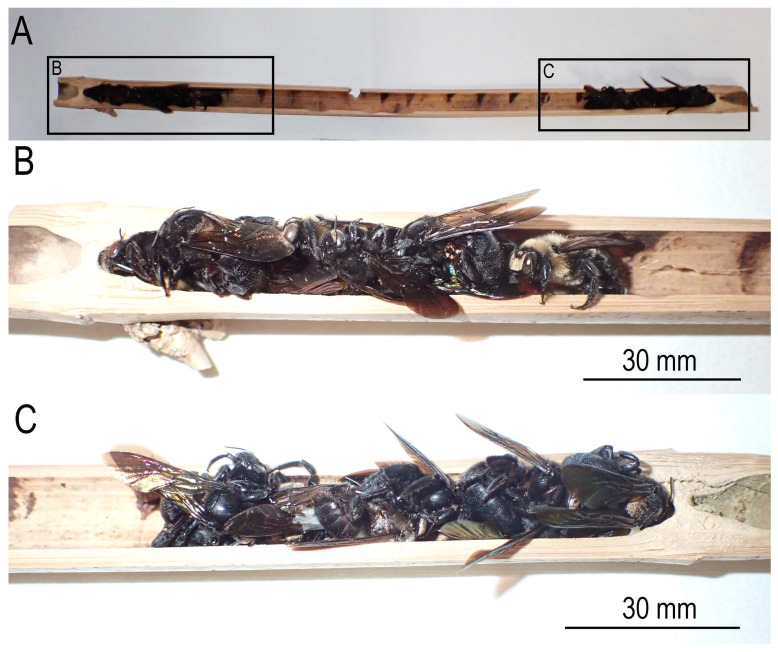
Hibernating adults of *Xylocopa* (*Biluna*) *tranquebarorum tranquebarorum*. (**A**) Transverse section of internal internode (lacuna) containing the bees; (**B**,**C**) close-up of both sides of the section. The bees are killed and dried specimens.

The bamboo carpenter bee, *Xylocopa* (*Biluna*) *tranquebarorum tranquebarorum* (Swederus), is widely distributed through the southern part of continental China to Taiwan [1] (Figure 1). The bee utilizes dead bamboo culm for its nesting, i.e., the bees bore a hole through the culm surface, enter, and use the empty or less dense internal internode (lacuna) for their nest (Figure 2). In contrast with typical carpenter bees (*Xylocopa* spp.), which cut long tunnels in dry wood [2,3,4,5], the nesting of the bee could be more cost-effective, i.e., the bee needs to bore only through the wall into an internal internode (lacuna) of the culm, and not bore a long tunnel in solid wood [6].

The bee invaded Japan around 2006 [7], and thereafter, its distribution has expanded rapidly through the central regions [7,8] where it causes damage to bamboo fencing of houses through nest establishment and its presence increases the potential for nuisance interactions with humans (i.e., sting injuries/reactions or fear of such events) [8,9]. In the Kyoto Prefecture, where the experimental sites in the present study are located, the bee invaded around 2014 [10].

In the beginning, the ecological and economic impacts of the bee were assessed as being relatively minor [8]. However, considering the fast pace of invasion [7,8], the ecological impact of the bee should be re-evaluated, e.g., the competition against its native relative, *X. appendiculata circumvolans*, for nectar and flower pollen, as well as the other native arthropods that utilize the bamboo for their nesting sites, should be examined. The biological information on this invasive bee has not been updated since Kawazoe [7] and Yamagishi et al. [8].

The general biology of the bee was originally examined by Iwata [6,11] and Maeta et al. [12,13] in its native ranges, continental China and Taiwan, respectively. According to these authors [6,11,12,13], the bee is an univoltine species. In spring, overwintered adults depart from their nest/hibernation site, mate, and females start to establish a new nest by boring a hole through a dead bamboo culm. The female usually lays about eight eggs, and each egg is enclosed with food (pollen mass) by a thin partition constructed from the inner surface fiber of the culm. The first four eggs enclosed on one side of the section develop into females, while the rest arranged on the other side of the section develop into males during the summer. Females develop to adults and emerge earlier than males, but because of their partitioning on the opposite side of the section, they do not damage males’ cells during emergence. Thereafter, newly eclosed females go out from the nest to feed on nectar to await development of their reproductive system (i.e., something akin to maturation feeding occurs). During this ‘maturation feeding’, females feed diurnally and return to their original nests at night. Sometimes bees will enter another mother’s nest at night, where they are usually not rejected by the residents of the nest. This maturation feeding lasts until autumn, and the bees overwinter in the nests.

He et al. [14,15] observed the nesting preference of *X. t. tranquebarorum* in continental China and reported that the bee preferred *B. multiplex* and *Phyllostachys* spp. culms with 12–25 mm in diameter, and sometimes utilized reed stems as well [14], or *B. multiplex* culms 17–21 mm in diameter in a different year of observation [15].

In Japan, Yamagishi [8] examined the basic biology of the bee in its invasive range, the central part of the main island of Japan. They reported that the bee prefers culms less than 30 mm in diameter, and sometimes more than eight (up to 13) bees can be found in a culm [8].

In their observations, the nesting preference of the bee was mostly focused on the thickness of the bamboo culms, and Iwata [6] noted that the bees prefer *Bambusa stenostachya*, which has a thick culm wall, allowing bees to re-use it for up to five years (seasons), and He et al. [14] mentioned the preferable host bamboo species. Further, these reports occurred mostly during the active (foraging and developing) seasons, i.e., spring to summer or autumn, and the details, e.g., number, sex ratio and condition of hibernating individuals, were not observed/reported sufficiently.

In addition, the biological characters of invasive species sometimes drift from those in their natural range during their adaptation to the invasive range [16,17]. For example, in the present case, *X. tranquebarorum*, which was previously reported as using only dead bamboo culm, was observed using fresh bamboo in Japan [18], possibly suggesting the behavioral plasticity of the bee in its invasive range.

Therefore, the potential ecological impact of this rapidly spreading bee needs to be evaluated based on its updated biological information, including its biological interaction with other arthropods sharing its nesting habitat.

In the present study, we investigated the nesting preference of the invasive bee using bamboo species collections in Kyoto, Japan, and examined the arthropods co-habiting with the bee in their nest and those re-using the old nest of the bee.

## 2. Materials and Methods

### 2.1. Morphometrics of Bee-Bored Sections and Co-Habiting Arthropods

The *X. t. tranquebarorum* colonized nesting materials were obtained from the bamboo species collection at the Kansai Research Center, Forestry and Forest Products Research Institute, where ca 40 species of bamboo are planted separately (Site 1, Table 1). From December 2023 to January 2024, damaged culms were collected during the management of the planted bamboo, e.g., during dead culm removal and pruning for density management.

Within the damaged culms, the sections bored by the bee were measured for their section length, diameter at the mid part of the section, and the relative position (s) of hole (s). Thereafter, the sectioned culms were split open, and presence/absence and the number of hibernating bees, the presence/absence of other arthropods in the section, and/or co-habiting with the bee and/or re-using the section bored in the previous year (s) were recorded. Simultaneously, the nesting process, e.g., presence/absence of scraping wounds on the internal surface and the trace of partition for larval/pupal chamber, were visually examined and recorded. Arthropods co-habiting with the bee or re-using the bored section were identified based on their morphological characters and recorded.

### 2.2. Host Preference of the Bee from Six Different Bamboo Collections

In addition to the first site, the nesting of the bee was surveyed visually at five other bamboo species collection sites in the Kyoto Prefecture. The information for each of the study sites is summarized in Table 1. Because the management procedure was already finished at the time, most dead culms had already been cut and removed. Thus, we walked through the bamboo collection and recorded any of the bamboo species bored by the bee. Therefore, we could observe only remaining dead culms in the present observation, i.e., the host range of the bee could be underestimated. The entrance hole of the bee was visually identified based on its size and shape, i.e., ca 4–5 mm diam., with mortar-shaped edge.

Because there are various opinions on the taxonomic status of bamboo species, we followed Suzuki [19] for bamboo scientific names rather than the facility records. For any species not listed in Suzuki [19], we provide only the genus and section name and cite the facility-recorded full name in the footnotes.

### 2.3. Statistical Analysis

The statistical analyses on the morphometrics of the culm and the numbers of hibernating bees were conducted using either the Chi-square test or ANOVA with Tukey–Kramer test. All analyses were performed using R version 4.4.3 [20].

## 3. Results

### 3.1. General Information for Bored Sections in Site 1

In site 1, 150 damaged sections were recovered and examined, and 7 species (8 varieties/forma) of bamboo out of ca 50 planted species were bored by the bee (Table 2). Within the 150 damaged internode sections, 99 (66%) were in dead culm harboring hibernating bees; 21 (14%) were in dead culm without the bee, i.e., bored in the previous year (s), and not used in this season; 9 (6%) were in dead culm without bee nest processing (abandoned after boring the internode) in the present or previous year (s); and the remaining 21 (14%) harvested sections were in fresh culm without any bee nest processing, i.e., bored in the present season but not utilized for nesting (Table 2). The original raw data for site 1 is provided in Supplementary Material S1.

**Figure 3:**
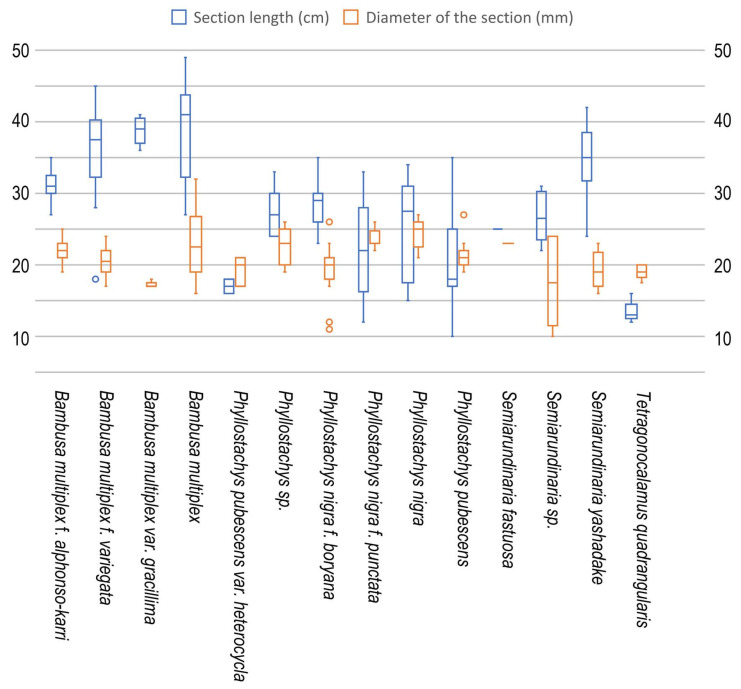
Length and diameter of the bee-damaged bamboo internode sections obtained for site 1.

Although the average internode length and thickness (diameter) varied among bamboo species, the total average length and thickness of bored sections were 29.8 ± 9.0 (10–49) cm and 21.2 ± 3.5 (10–32) mm, respectively (Figure 3, Table 3). Although the section length was highly variable, the external diameter was mostly between 16 and 28 mm. The entrance of the nest (bored hole) was located at 10.7 ± 9.4 (1–38) cm, which was 33.2 ± 25.1 (2.3–96.3)% of section length from the bottom of the internode section (Table 3).

### 3.2. Observation in Sites 2–7

The observations are summarized in Table 4.

In sites 2–7, 15 species (further separated into 29 taxon) were recognized to be damaged by the bee. Within these 15 species, *Bambusa multiplex* (original, form *alphonso-karri*, form *variegata*) and *Phyllostachys* spp. were frequently utilized by the bee.

Because nesting attempts were only externally observed in sites 2–7, the following life history results are all from site 1.

### 3.3. Sections Harboring Hibernating Bees

The hibernating bees were confirmed in 99 sections consisting of 11 species (13 forma/varieties/cultivars) (Table 5). The average length and thickness were 29.8 ± 10.3 (10–49) cm and 21.2 ± 3.4 (12–32) mm, respectively, and the entrance holes were bored at 11.4 ± 10.0 (1–38) cm, or 34.6 ± 25.0 (2.3–92.3)% of section length from bottom of the internode section (Table 5).

The average internode length and external diameter varied among bamboo species from 13.4 ± 1.5 (12–16) cm (*T. quadrangularis*) to 39.0 ± 6.7 (27–49) cm (*B. multiplex*), and 17.2 ± 0.4 (17–18) mm (*B. multiplex* var. *gracillima*) to 25.5 ± 1.0 (24–27) mm (*P. nigra* var. *nigra*), respectively. Statistically, the average internode section length was significantly longer in *B. multiplex*, and shorter in *P. nigra* var. *nigra*, *P. pubescens*, and *T. quadrangulari*; internode section diameter was thicker in *P. nigra* var. *nigra*, and thinner in *B. multiplex* var. *gracillima*, *P. nigra* f. *boryana*, and *T. quadrangulari*. These variations probably reflect the natural range of each bamboo species/forma/variety/cultivar. The position of the entrance hole was relatively high in *B. multiplex f. variegata*, 45.9 ± 17.5 (14.3–69.0)% from the bottom of the internode, and *B. multiplex* var. *gracillima*, 45.0 ± 11.5 (30.8–57.9)%, and low in two cultivars of *P. nigra*, 5.0 ± 1.6 (3.1–8.3)% and 6.9 ± 4.7 (3.3–14.3)%, but the variation within and among species was high, and no clear tendency was observed between the position and the internode section length/thickness (Table 5).

**Figure 4:**
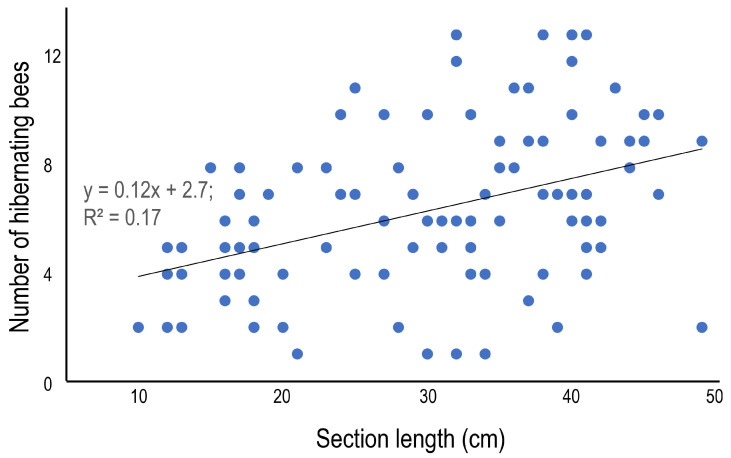
Relationship between the length and the number of hibernating bees in the internode section at site 1.

**Figure 5:**
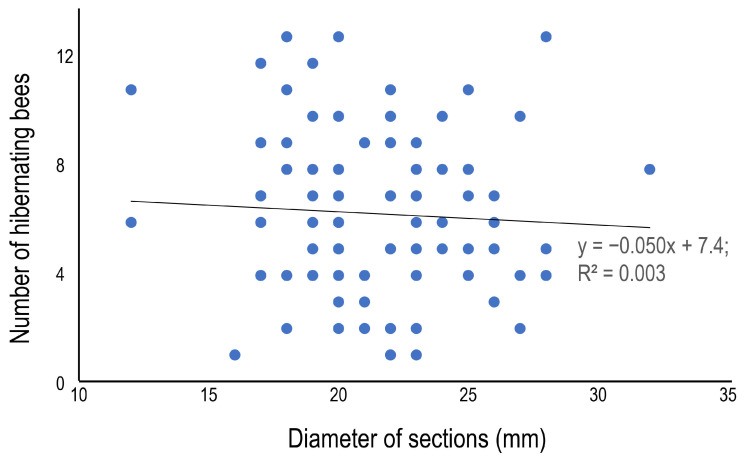
Relationship between the diameter and the number of hibernating bees in the internode section at site 1.

The average number of hibernating bees was 6.3 ± 3.1 (1–13), and although some variation among the bamboo species was observed—the number was statistically higher in *B. multiplex* 8.2 ± 3.5 (1–13), and lower in *P. pubescens* 4.9 ± 2.0 (2–8)—there was no clear difference among bamboo species (Table 5). In terms of the morphometrics of the bamboo sections, the section length (Figure 4) and the diameter (Figure 5) are presented as scatter plots. Section length was positively correlated with the number of hibernating bees, but diameter was not (*p* = 0.05, regression analysis). The positive correlations could be derived from the relatively large sample number (=99). The average sex ratio (% of females to total bees) was 62.7%, and the ratio varied from 50.0 to 83.3% among bamboo species, but significant variation was not detected among bamboo species (Table 5).

### 3.4. Previously Used Sections Without Hibernating Bees

Within 150 bored sections, 21 (14%) sections comprising six species did not harbor hibernating bees, but had scraping wounds on the internal surface and the trace partitioning for larval/pupal chambers, i.e., these sections were utilized by the bee in the previous year (s) but were not used in the present season (Table 2 and Table 6). The average length and diameter of the sections were 31.9 ± 4.8 (24–42) cm and 20.5 ± 4.5 (10–30) mm, respectively, and the entrance holes were bored at 9.7 ± 8.1 (1–31) cm, or 30.4 ± 23.9 (2.4–79.5)% of section length from bottom. Probably because of the large variation, the length, diameter and the position of the entrance holes were not statistically different from ones harboring the hibernating bees in the present season at site 1 (Table 5 and Table 6).

Although these internode sections did not harbor the bee, some had prepupae of *Anterhynchium gibbifrons* Yamane & Murota (Hymenoptera), adults of *Dinoderus japonicus* Matsumura (Coleoptera), unidentified moth larvae, and some other arthropods as secondary users of the nest (Table 7). Further details are mentioned below.

### 3.5. Other Sections

The remaining 30 of the 150 (20%) sections from site 1 were bored by the bee, but no nest processing was observed. Within these 30, 9 and 21 were dead and fresh culms, respectively (Table 2, Table 8 and Table 9). The section length and diameter, and the position of bee bored holes are as follows: 26.1 ± 9.4 (13–41) cm and 21.2 ± 5.1 (11–26) mm 4.9 ± 6.0 (1–18) cm = 19.2 ± 21.3 (2.4–55.6)% from bottom for dead culm; 29.3 ± 3.7 (22–37) cm and 22.2 ± 1.6 (19–25) mm, and 10.8 ± 8.4 (1–26) cm and 22.2 ± 1.6 (19–25) mm, and 10.8 ± 8.4 (1–26) cm = 36.2 ± 28.0 (2.7–96.3)% from bottom for fresh culm (Table 8 and Table 9). Some of them contained arthropods as secondary users, and further details will be mentioned below.

### 3.6. Arthropods Co-Habiting with the Carpenter Bee

In addition to *X. t. tranquebarorum*, several other arthropods were recognized as co-habitants and/or secondary users of the bored sections. *A. gibbifrons*, *D. japonicus*, *Crematogaster matsumurai* Forel (Hymenoptera), unidentified moth larvae, and an unidentified spider were found in currently or previously used sections (Table 7). Meanwhile, *A. gibbifrons*, unidentified moth larvae, an unidentified spider, and an unidentified shield bug were recognized from the section without bee nest processing (Table 10). In some cases, these arthropods co-habited with the bee or other arthropods, i.e., *D. japonicus* co-habited with the bee, *A. gibbifrons*, and *C. matsumurai*, and moth larvae and spider were found from the same section (Table 7 and Table 10).

**Figure 6:**
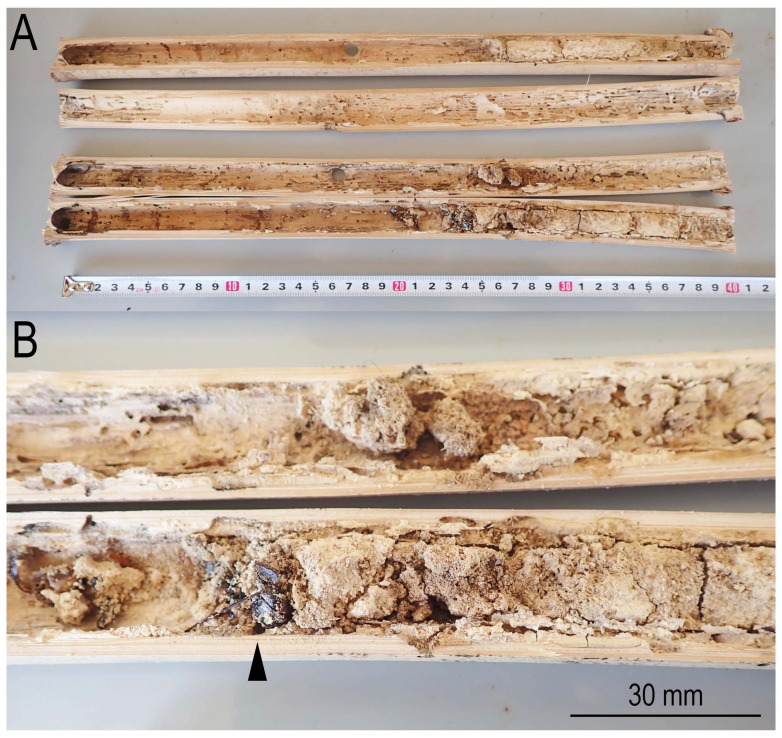
Internode filled by *Dinoderus japonicus* frass. (**A**) Transverse sections; (**B**) close-up of the internode section filled by the frass. A bee covered by the frass is indicated with an arrowhead.

Within these co-habitants, in cases of heavy infestation of *D. japonicus*, the internal hollow of the culm was stuffed by the beetle frass (Figure 6), and the hibernating bees were covered or buried by the frass.

### 3.7. Additional Observation

**Figure 7:**
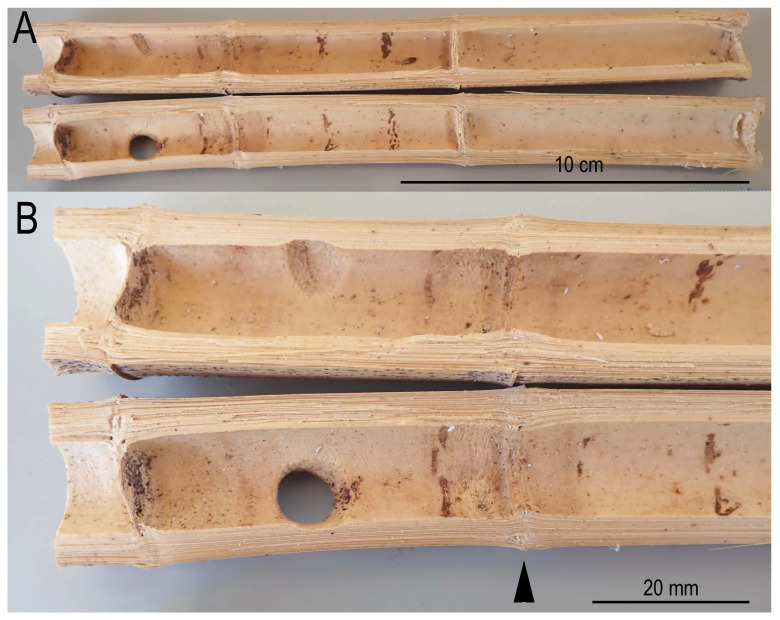
The bee removed a septa wall (diaphragm) between two short internode sections to utilize them as an expanded nest volume. (**A**) Transverse section of three internodes; (**B**) close-up of node (arrowhead) where the diaphragm was removed by the bee to expand into the adjacent internode.

As an additional observation, *X. t. tranquebarorum* entered a very short (5 cm) internode of *P. pubescens*, and then opened the upper adjacent septa (7 cm) and connected them to form a 12 cm long nest (Figure 7).

**Figure 8:**
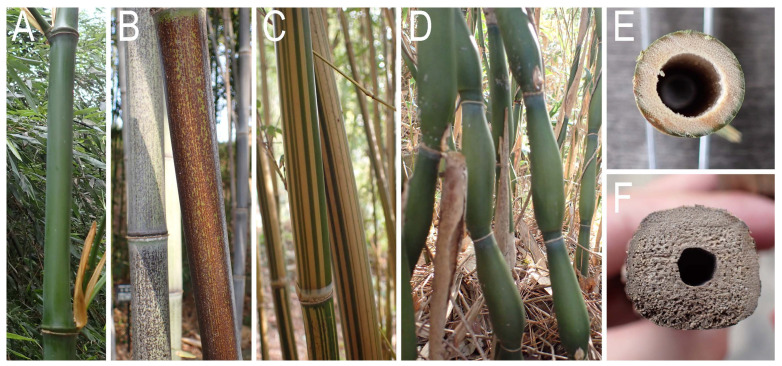
Culm morphology of several bamboo species. (**A**) *Phyllostachys bambusoides* with green culm without special pattern. (**B**) *P. nigra* f. *punctata* with brownish surface. (**C**) *Bambusa multiplex* f. *alphonso-karri* with striped pattern on culm surface. (**D**) *Pseudosasa japonica* var. *tsutsumiana* culm consisting of elongate pear-shaped sections. (**E**) Circular cross-section of *P. bambusoides* culm. (**F**) Cross-section of *Tetragonocalamus quadrangularis* culm with roundish square shape.

Concerning the color, surface pattern and shape of culm, the bees bored *P. nigra* forma and varieties with a brown surface, *B. multiplex* f. *alphonso-karri* with a striped pattern, and *T. quadrangularis* with a somewhat squared cross-section (Figure 8). In addition, although the nesting was not observed in the examined season, *Pseudosasa japonica* var. *tsutsumiana* Yanagita, which has elongated and pear-shaped internodes (Figure 8), were also bored by the bee (Kanzaki, unpubl. obs.).

## 4. Discussion

### 4.1. Host Preference of the Bee

Field observations in the present study revealed that the bamboo carpenter bee, *X. t. tranquebarorum*, utilized a total of 18 species (31 forma/varieties/cultivars) in seven bamboo species collections in Kyoto, Japan (Table 2 and Table 4). As far as we know, this is the first case of a large-scaled survey of host nesting preference (potential host range) of the bee.

In the present study, the bees did not show clear host preference, but the external diameter of the internode, approximately 21.2 ± 3.5 mm, appeared to be an important nesting parameter. Also, the color and surface pattern of the culm did not seem to affect the bees’ host preference, i.e., a culm with dark color (*P. nigra* forma/varieties), striped pattern on the surface (*B. multiplex* f. *alphonso-karri*), and/or squared cross-section of the internodes (*T. quadrangularis*) were also utilized by the bee. In addition, the bee attempted to bore the culm composed of elongate pear-shaped internode sections (*P. japonica* var. *tsutsumiana*: Kanzaki pers. obs.), live culm ([18]; present study), wood sticks (Kanzaki pers. obs.), and reeds [14]. Thus, the potential host range of the bee does not seem to be limited to only the dead culms of bamboo.

So far, the host preference of the bee has not been examined closely in its natural range. Therefore, although the host range partially overlapped with that in continental China [14,15], applicability of the present findings to their native range has not been confirmed. In other words, the species might have expanded its host preference adapting to the range of bamboo species available in Japan, or the insufficient amount of available resources might have decreased the strictness of their host preference, e.g., they attempted to nest in fresh bamboo [18] or wood sticks, which has not been reported previously. Considering the present situation, the adaptability of this bee to a new environment seems quite high.

As an additional observation, two short (5 cm and 7 cm) internal internodal sections (lacuna) were connected as a 12 cm-long nest section by removing the internal partition (septa or diaphragm) between the sections (Figure 7). This nesting pattern of the bee has not been reported previously, and thus these behaviors could be rare cases, possibly occurring due to a shortage of available nesting resources.

The rather low strictness of the host nesting preference confirmed in the present study suggests that the bee could damage a wider range of materials than currently reported.

### 4.2. Number and Sex Ratio of Hibernating Bees

In the present study, the average number of hibernating bees in a positive internode was 6.3 ± 3.1, and the number varied between 1 and 13. The average sex ratio was ca 63%, i.e., male–female ratio was approximately 1:2. These averages seem partially concordant with the observation by Iwata [6], i.e., eight progenies with 1:1 sex ratio, and Maeta et al. [13] and Yamagishi et al. [8], who reported, 6 ± 1 (4–8) and 6.2 in average brood cells per section, respectively. Bees emerge within the season, and thus it takes a couple of months between emergence and hibernation. Thus, if the initial sex ratio was 1:1 as suggested in the previous study, male mortality within the period might be higher than female. Yamagishi et al. [8] noted that the sex ratio varies among seasons, and Kishi [10] also noted a female-biased sex ratio in a Japanese population, although the detailed mechanism for the variation was not clarified. Further continuous observation would be necessary to understand the hibernation characters of the bee in native and invasive ranges, e.g., seasonal change of the sex ratio, in native and invasive ranges would need to be compared to verify the hypothesis.

The variation in the number of bees per internode, 1–13 in the present study and sometimes up to more than 30 (Kanzaki, unpubl. obs.), does not fit the previous observations. Iwata [6] reported that the progeny bees exhibited foraging behavior to obtain nutrients for their hibernation and then returned to their mother nest to hibernate. Therefore, the maximum number found in the present study, 13, is obviously higher than the common number of the progenies, 8 [6] or 6 ± 1 (4–8) [13], suggesting that the progenies do not necessarily hibernate in their mother’s nest. Although Iwata [6] noted that the bees sometimes enter the wrong nest, this also occurs in the hibernation. Interestingly, Yamagishi et al. [8] also reported that up to 13 bees were hibernating in a section in Japan. Thus, the larger numbers of hibernating bees in a section could be a character occurring in the invasive population. Because detailed information concerning their hibernating behavior has not been reported, it is not clear whether this entrance into the wrong nest or aggregation during hibernation commonly occurs in their native range or not. However, this behavior could be adaptive. For example, aggregations of bees from multiple nests could improve the opportunity of outcrossing for the bee, and/or help in moderating their temperature during hibernation in Japan to be cooler than their native range. Further field observation in their native and invasive ranges as well as analysis of genetic structure, i.e., the basis of such “social behavior”, is necessary to understand their hibernation behaviors.

### 4.3. Co-Habitant and Secondary User Arthropods

Except for an examination of an entomophilic mite [9], the direct biological interaction between the bee and other arthropods had not been examined previously. Meanwhile, the present study revealed that various arthropods were associated with the bee as a co-habitant or secondary user of the nest.

Within these arthropods, an ant (*C. matsumurai*), unidentified shield bug, and spider were not frequently found, and their interaction with the bee, e.g., competition or predation, was not clear. Meanwhile, lepidopteran larvae, *A. gibbifrons* and *D. japonicus* were found relatively frequently.

Lepidopteran larvae were found as both a co-habitant and a secondary user. As a co-habitant, the number of hibernating bees in the internode was 4.6 ± 3.8 (0–8) (Table 6), which is not significantly different from the average number of hibernating bees in an internode section, 6.3 ± 3.1 (1–13) (Table 4), suggesting that the larvae and the bee are not directly interacting, although both the larvae and the bees utilize the same part of the internode section (lacuna) for their hibernation.

*Anterhynchium gibbifrons* also shares the lacuna with the bee. Interestingly, the average number of bees co-habiting with *A. gibbifrons*, 0.25 ± 0.7 (0–2) (Table 6), is smaller than the total average 6.3 ± 3.1 (1–13) (Table 4), and also smaller than the number co-habiting with lepidopteran larvae, 4.6 ± 3.8 (0–8) or *D. japonicus*, 8.2 ± 3.3 (0–13) (Table 6), suggesting that the wasp negatively affects the nesting of the bee. However, the process of their interaction and the mechanism of the negative effect(s) are unknown. The wasp was first found in Fukui, Japan [21], and successively found in various areas in Japan [22]. According to this pattern, although its geographical origin has not been determined [23,24], the wasp is currently considered as an invasive/introduced species to Japan, because such a conspicuous wasp species had not been found in Japan before 2007 [21]. The wasp is reported as a monophagous species in Japan, feeding only on the larvae of a moth, *Demobotys pervulgalis* [24]. Therefore, the wasp likely does not predate the bee (larvae or pupae) in the nest, and they could simply compete against the bee for their nest site and eliminate the resident bees during their nesting. Because both the bee and the wasp are invasive species to Japan, their interaction could be a case of that between invasive species sharing or competing for the same nesting resource in their invasive range. Further chronological observation of their interactions in the field would be necessary to evaluate their ecological relationships.

In contrast, the powder-post beetle, *D. japonicus*, utilizes mostly the wall of the bamboo culm [25]. Therefore, they can utilize the same bamboo culm independently, i.e., they are segregating each other by the usage of the culm, and the average number of hibernating bees in the internode infected by *D. japonicus*, 8.2 ± 3.3 (0–13), was not significantly different from the average. However, in the present observation, the lacuna was sometimes totally filled by the beetle frass and the bees were buried by it (Figure 6). In addition, the beetles’ feeding weakened the culm structurally. Thus, the utilization by the beetles possibly prevents the bees’ continuous utilization of the culm. Therefore, long-term field observation is necessary to evaluate their ecological interaction more precisely.

## 5. Conclusions

As an invasive species, the bamboo carpenter bee, *X. t. tranquebarorum*, should be monitored and eradicated. Based on the currently available information, i.e., the bee hibernates in its nest in dead bamboo culms, we posit that hibernating individuals could be the primary target for eradication because the bees are inactive, i.e., the risk of stinging injury is lower, and it is relatively simple to cut and burn bee-infested culm. However, only a few studies have been conducted on the general biology of the bee in its native and invasive range, and therefore, it is important to understand their hibernation behavior more completely for the development of an effective eradication strategy for the bee.

In the present study, we examined the nesting preference and co-habitants in the hibernation nest of the bee. As such, two important points were revealed; (1) the host preference (specificity) of the bee is rather low, and several bamboo species with atypical culm shape and color could also be the host, suggesting that the potential host range is much wider than previously reported; and (2) several co-habitants could negatively affect the bee, suggesting that further field observations could reveal possible suppressors of the bee. Particularly, the other invasive species, *A. gibbifrons*, and the bee seemed to be competitors for the same nesting resources. Considering the present status of worldwide trading, biological invasions are occurring in many different places, and the relationship between the bee and the wasp could be an interesting case of possible negative interactions between two (or more) invasive species.

Currently, little is known about the bamboo carpenter bee. Therefore, further field observations on the species, especially on its behavior during the active season and concerning interacting organisms [9,26], are necessary in its native and invasive ranges.

## Figures and Tables

**Table 1:** List of study sites.

Site Number	Place	Locality	Collection Date or Period	GPS
1	Kansai Research Center, Forestry and Forest Products Research Institute	Fushimi, Kyoto, Japan	December 2023 to January 2024	34°56′29″ N, 135°46′21″ E; 50 m a.s.l.
2	Kamigamo Experimental Station of Kyoto University	Sakyo, Kyoto, Japan	16 February, 19 December 2024	35°04′12″ N, 135°45′53″ E, 141 m a.s.l.
3	Botanical Garden of the Graduate School of Science	Sakyo, Kyoto, Japan	19 December 2024	35°01′48″ N, 135°47′14″ E, 72 m a.s.l.
4	Kyoto Botanical Gardens	Sakyo, Kyoto, Japan	19 December 2024	35°02′58″ N, 135°45′54″ E, 71 m a.s.l.
5	Rakusai Bamboo Park	Nishikyo, Kyoto, Japan	19 December 2024	34°57′34″ N, 135°41′12″ E, 85 m a.s.l.
6	Oomoto Kameyama Botanical Park	Kameoka, Kyoto, Japan	13, December 2024	35°00′49″ N, 135°34′55″ E, 100 m a.s.l.
7	Shokado Garden	Uji, Kyoto, Japan	20, December 2024	34°51′48″ N, 135°42′15″ E, 17 m a.s.l.

**Table 2:** Summary of examined *X. t. tranquebarorum* bored bamboo internode sections from site 1.

Bamboo Species/Variety	Dead Internode with Overwintering Bees	Dead Internode Without Overwintering Bees	Dead Internode Without Bee Nest Processing	Live Internode Without Bee Nest Processing
*Bambusa multiplex* Raeuschel f. *alphonso-karri* Nakai	2	2	0	9
*B. multiplex* Raeuschel f. *variegata* Hatusima	15	2	1	0
*B. multiplex* Raeuschel var. *gracillima* Sad. Suzuki	5	0	0	0
*B. multiplex* Raeuschel	23	1	0	0
*Phyllostachys pubescens* Mazel ex Houzeau de Lehaie var. *heterocycla*	3	0	0	0
*Phyllostachys* sp. ^1^	1	0	1	5
*P. nigra* f. *punctata* Nakai	10	0	2	0
*P. nigra* Munro var. *nigra*	6	5	0	0
*P. nigra* Munro f. *boryana* Makino	8	3	2	0
*P. pubescens* Mazel ex Houz. de Leh.	13	0	1	1
*Semiarundinaria*. sp. ^2^	1	2	0	3
*S. fastuosa* Makino var. fastuosa	0	0	0	1
*S. yashadake* Makino	7	6	2	2
*Tetragonocalamus quadrangularis* Nakai	5	0	0	0
Total	99	21	9	21

^1^ The signboard displayed the Japanese and scientific names “*Hime-hachiku*” and *Phyllostachys humilis* Muroi, respectively. ^2^ The signboard displayed the Japanese and scientific names “*Kenashi-narihira*” and *Semiarundinaria fastuosa* Makino, respectively.

**Table 3:** Length, bee-bored positioning (%) and thickness of all examined internode sections from site 1.

Bamboo Species/Variety	N	Section Length (cm)	Bore Hole Position (cm from Bottom)	Bore Hole Position (% from Bottom)	Diameter at the Middle (mm)
*Bambusa multiplex* f. *alphonso-karri*	13	31.1 ± 2.1 (27–35)	18.0 ± 3.9 (12–26)	58.6 ± 14.7 (38.7–96.3)	22.0 ± 1.8 (19–25)
*B. multiplex* f. *variegata*	18	35.9 ± 6.6 (18–45)	16.3 ± 9.0 (1–31)	43.2 ± 18.9 (3.0–69.0)	20.7 ± 1.9 (17–24)
*B. multiplex* var. *gracillima*	5	38.8 ± 1.9 (36–41)	17.4 ± 4.3 (12–22)	45.0 ± 11.5 (30.8–57.9)	17.2 ± 0.4 (17–18)
*B. multiplex*	24	39.1 ± 6.5 (27–49)	18.5 ± 9.3 (1–38)	45.6 ± 19.5 (2.3–82.6)	23.0 ± 4.4 (16–32)
*Phyllostachys pubescens* var. *heterocycla*	3	18, 17, 16	1, 2, 1	5.8, 11.8, 6.3	21, 20, 17
*Phyllostachys* sp. ^1^	7	27.4 ± 3.4 (24–33)	7.9 ± 4.0 (1–14)	29.6 ± 16.1 (3.3–56.0)	22.7 ± 2.6 (19–26)
*P. nigra* f. *punctata*	12	30.6 ± 6.9 (12–33)	10.9 ± 0.3 (1–2)	32.0 ± 1.6 (3.1–8.3)	21.1 ± 1.3 (22–26)
*P. nigra* var. *nigra*	11	25.2 ± 6.9 (15–34)	7.8 ± 6.1 (1–18)	30.7 ± 23.5 (2.9–66.7)	24.3 ± 2.0 (21–27)
*P. nigra* f. *boryana*	13	28.2 ± 4.3 (18–35)	3.0 ± 3.2 (1–10)	11.7 ± 15.4 (3.3–55.6)	19.2 ± 4.0 (11–26)
*P. pubescens*	15	20.5 ± 7.5 (10–35)	9.9 ± 8.4 (2–29)	46.3 ± 28.7 (11.1–92.3)	21.3 ± 1.9 (19–27)
*Semiarundinaria* sp. ^2^	6	26.7 ± 3.4 (22–31)	3.7 ± 4.3 (1–12)	13.8 ± 15.9 (3.3–44.4)	17.5 ± 6.8 (10–24)
*S. fastuosa* var. *fastuosa*	1	25	1	4	23
*S. yashadake*	17	34.3 ± 5.5 (24–42)	6.4 ± 9.3 (1–31)	17.8 ± 23.8 (2.4–79.5)	19.3 ± 2.3 (16–23)
*Tetragonocalamus quadrangularis*	5	13.4 ± 1.5 (12–16)	4.4 ± 3.4 (1–10)	33.5 ± 26.3 (7.7–76.9)	18.2 ± 0.8 (17–19)
Total	150	29.8 ± 9.0 (10–49)	10.7 ± 9.4 (1–38)	33.2 ± 25.1 (2.3–96.3)	21.2 ± 3.5 (10–32)

The values are in the form mean ± SD (range) for the materials with n = 5 or more, and actual values are given for the materials with n = 4 or less. ^1^ The signboard displayed the Japanese and scientific names “*Hime-hachiku*” and *Phyllostachys humilis* Muroi, respectively. ^2^ The signboard displayed the Japanese and scientific names “*Kenashi-narihira*” and *Semiarundinaria fastuosa* Makino, respectively.

**Table 4:** List of bamboo species damaged by the bee in sites 2–7.

Damaged Bamboo Species	Sites Found
*Bambusa multiplex*	4, 5
*B. multiplex* f. *alphonso-karri*	2, 4, 5
*B. multiplex* f. *variegata*	4, 5, 7
*Phyllostachys aurea* ^1^	2, 5
*P. aurea* var. *flavescens-inversa*	2
*P. bambusoides*	2
*P. bambusoides* f. *subvariegata*	2
*P. bambusoides* var. *marliacea*	5
*P. bambusoides* var. *castillonis*	2, 5
*P. makinoi*	2
*P. nigra* var. *nigra*	7
*P. nigra* f. *boryana*	2, 3, 4
*P. nigra* f. *megurochiku*	2
*P. nigra* f. *punctata*	2, 4, 5
*P. nigra* var. *henonis*	2
*P. nigra* var. *tosaensis*	5
*P. pubescens*	2
*P. pubescens* var. *heterocycla*	2, 5
*P. pubescens* var. *nabeshimana*	7
*P. vissetii*	5
*Phyllostachys* sp. ^2^	5
*Pleioblastus* sp. (Subgen. *Pleioblastus*)	3, 4, 6
*P*. *hindsii*	5
*Pseudosasa japonica*	5
*Semiarundinaria fastuosa*	4
*S*. *fastuosa* var. *viridis*	5
*Sinobambusa tootsik*	3, 5
*Tetragonocalamus quadrangularis*	3

^1^ The signboard displayed the Japanese name “Ougon-hotei,” referring to a variety of *Phyllostachys aurea*, whose culm is entirely yellow. ^2^ The signboard displayed the Japanese and scientific names “Hime-hachiku” and *Phyllostachys humilis* Muroi, respectively.

**Table 5:** Length, bee-bored entrance hole position and external diameter of dead internode sections harboring hibernating *Xylocopa t. tranquebarorum* with number and sex ratio of bees from site 1.

Bamboo Species/Variety	n	Section Length (cm)	Bore Hole Position (cm)	Bore Hole Position (%)	Diameter (mm)	Number of ♂ Bees	Number of ♀ Bees	Total Bees	Sex Ratio (♀%)
*Bambusa multiplex* f. *alphonso-karri*	2	30, 33	13, 20	43.3, 60.6	22, 25	0, 1	1, 4	1, 5	83.3
*B. multiplex* f. *variegata*	15	36.5 ^ab^ ± 7.1 (18–45)	17.6 ± 8.9 (4–31)	45.9 ^a^ ± 17.5 (14.3–69.0)	20.9 ^bcd^ ± 2.0 (17–24)	2.7 ± 2.1 (0–6)	4.3 ± 2.3 (0–8)	7.0 ^ab^ ± 3.2 (2–13)	61.0
*B. multiplex* var. *gracillima*	5	38.8 ^ab^ ± 1.9 (36–41)	17.4 ± 4.3 (12–22)	45.0 ^a^ ± 11.5 (30.8–57.9)	17.2 ^d^ ± 0.4 (17–18)	2.2 ± 1.3 (1–4)	5.4 ± 1.9 (3–8)	7.6 ^ab^ ± 2.9 (4–12)	72.2
*B. multiplex*	23	39 ^a^ ± 6.7 (27–49)	18.5 ± 9.5 (1–38)	45.7 ^a^ ± 19.8 (2.3–82.6)	22.7 ^abc^ ± 4.3 (16–32)	3.0 ± 1.6 (0–7)	5.2 ± 2.7 (0–11)	8.2 ^a^ ± 3.5 (1–13)	62.9
*Phyllostachys pubescens* var. *heterocycla*	3	18, 17, 16	1, 2, 1	5.6, 11.8, 6.3	21, 20, 17	1, 2, 1	1, 6, 5	2, 8, 6	75.0
*Phyllostachys* sp. ^1^	1	27	7	25.9	19	2	2	4	50.0
*P. nigra* f. *punctate*	10	23.2 ^cd^ ± 6.9 (12–33)	1.1 ± 0.3 (1–2)	5.0 ^b^ ± 1.6 (3.1–8.3)	23.5 ^ab^ ± 1.1 (22–26)	2.2 ± 2.0 (0–7)	3.4 ± 2.2 (0–8)	5.6 ^ab^ ± 3.2 (1–10)	60.7
*P. nigra* var. *nigra*	6	19.2 ^d^ ± 4.0 (15–25)	6.2 ± 5.3 (1–12)	31.0 ^ab^ ± 26.4 (5.3–66.7)	25.5 ^a^ ± 1.0 (24–27)	2.7 ± 0.8 (1–3)	3.3 ± 1.5 (1–5)	6.0 ^ab^ ± 1.5 (4–8)	55.6
*P. nigra* f. *boryana*	8	29.6 ^bc^ ± 3.0 (25–35)	2.1 ± 1.6 (1–5)	6.9 ^b^ ± 4.7 (3.3–14.3)	19.0 ^d^ ± 3.0 (12–22)	2.3 ± 1.2 (0–4)	4.3 ± 2.1 (1–8)	6.5 ^ab^ ± 3.1 (1–11)	65.4
*P. pubescens*	13	19.4 ^d^ ± 7.3 (10–35)	10.0 ± 8.7 (2–29)	48.7 ^a^ ± 29.2 (11.1–92.3)	21.2 ^bcd^ ± 2.1 (19–27)	2.2 ± 1.3 (1–5)	2.8 ± 1.4 (1–5)	4.9 ^b^ ± 2.0 (2–8)	56.3
*Semiarundinaria* sp. ^2^	1	31	3	9.7	12	2	4	6	66.7
*S. fastuosa* var. fastuosa	0	-	-	-	-	-	-	-	-
*S. yashadake*	7	33.4 ^ab^ ± 4.9 (24–45)	7.7 ± 10.0 (1–31)	21.1 ^ab^ ± 25.4 (2.9–71.8)	19.0 ^cd^ ± 2.6 (16–24)	1.7 ± 1.2 (1–6)	3.4 ± 2.0 (0–8)	5.1 ^ab^ ± 2.7 (1–13)	66.7
*Tetragonocalamus quadrangularis*	5	13.4 ^d^ ± 1.5 (12–16)	4.4 ± 3.4 (1–10)	33.5 ^ab^ ± 26.3 (7.7–76.9)	18.2 ^d^ ± 0.8 (17–19)	1.4 ± 0.5 (1–2)	2.6 ± 0.9 (1–3)	4.0 ^ab^ ± 1.2 (2–5)	65.0
Total	99	29.8 ± 10.3 (10–49)	11.4 ± 10.0 (1–38)	34.6 ± 25.0 (2.3–92.3)	21.2 ± 3.4 (12–32)	2.4 ± 1.6 (0–7)	4.0 ± 2.2 (0–11)	6.3 ± 3.1 (1–13)	62.7

The values are in the form mean ± SD (range) for the materials with n = 5 or more, and actual values are given for the materials with n = 4 or less. Values with different characters in each column are significantly different (*p* = 0.05; ANOVA; Tukey–Kramer test). The treatments with n = 4 or less were excluded from statistical analysis. ^1^ The signboard displayed the Japanese and scientific names “*Hime-hachiku*” and *Phyllostachys humilis* Muroi, respectively. ^2^ The signboard displayed the Japanese and scientific names “*Kenashi-narihira*” and *Semiarundinaria fastuosa* Makino, respectively.

**Table 6:** Length, bee-bored position and external diameter of dead internode sections without harboring hibernating *Xylocopa t. tranquebarorum* (utilized in the previous season (s)) from site 1.

Bamboo Species/Variety	n	Section Length (cm)	Bore Hole Position (cm from Bottom)	Bore Hole Position (% from Bottom)	Diameter at the Middle (mm)
*Bambusa multiplex* f. *alphonso-karri*	2	29, 34	(15, 21) ^1^, 17	(51.7, 72.4) ^1^, 50.0	24, 23
*B. multiplex* f. *variegata*	2	33, 30	1, 11	3.0, 36.7	19, 19
*B. multiplex* var. *gracillima*	0	-	-	-	-
*B. multiplex*	1	42	18	42.9	30
*Phyllostachys pubescens* var. *heterocycla*	0	-	-	-	-
*Phyllostachys* sp. ^2^	0	-	-	-	-
*P. nigra* f. *punctate*	0	-	-	-	-
*P. nigra* var. *nigra*	5	31.2 ± 1.5 (30–34)	9.3 ± 6.9 (1–18)	30.5 ± 22.7 (2.9–58.1)	23.2 ± 2.1 (21–26)
*P. nigra* f. *boryana*	3	30, 30, 29	1, 1, 9	3.3, 3.3, 31.0	23, 20, 17
*P. pubescens*	0	-	-	-	-
*Semiarundinaria* sp. ^3^	2	27, 24	12, 4	44.4, 16.7	12, 10
*S. fastuosa* var. *fastuosa*	0	-	-	-	-
*S. yashadake*	6	35.0 ± 6.8 (24–42)	8.5 ± 11.40 (1–31)	23.7 ± 28.4 (2.4–79.5)	19.0 ± 1.46 (17–20)
*Tetragonocalamus quadrangularis*	0	-	-	-	-
Total	21	31.9 ± 4.8 (24–42)	9.7 ± 8.1 (1–31)	30.4 ± 23.9 (2.4–79.5)	20.5 ± 4.5 (10–30)

The values are in the form mean ± SD (range) for the materials with n = 5 or more, and actual values are given for the materials with n = 4 or less. ^1^ Two nesting holes presented in the same section. ^2^ The signboard displayed the Japanese and scientific names “*Hime-hachiku*” and *Phyllostachys humilis* Muroi, respectively. ^3^ The signboard displayed the Japanese and scientific names “*Kenashi-narihira*” and *Semiarundinaria fastuosa* Makino, respectively.

**Table 7:** Arthropods secondarily utilizing or co-habiting with *Xylocopa t. tranquebarorum* in the internode sections with evidence of bee nest processing at site 1.

Bamboo Species/Variety	*A. gibbifrons*	*A. gibbifron* + *D. japonicus*	*D. japonicus*	*D. japonicus* + *C. matsumurai*	*C. matsumurai*	Lepidopteran Larvae	Spiders
*Bambusa multiplex* f. *alphonso-karri*	1	-	-	-	-	1	-
*B. multiplex* f. *variegata*	1	1	7	1	1	-	-
*B. multiplex*	1	-	9	-	-	-	-
*Phyllostachys* sp. ^1^	-	-	-	-	-	1	-
*P. nigra* f. *punctata*	-	-	-	-	-	1	-
*P. nigra* var. *nigra*	-	-	-	-	-	1	-
*P. nigra* f. *boryana*	-	-	-	-	-	-	1
*Semiarundinaria* sp. ^2^	-	-	-	-	-	1	-
*S. yashadake*	5	-	-	-	-	-	-
Number of hibernating bees	0.25 ± 0.7 (0–2)	0	8.2 ± 3.3 (0–13)	7	8	4.6 ± 3.8 (0–8)	0

^1^ The signboard displayed the Japanese and scientific names “*Hime-hachiku*” and *Phyllostachys humilis* Muroi, respectively. ^2^ The signboard displayed the Japanese and scientific names “Kenashi-narihira” and *Semiarundinaria fastuosa* Makino, respectively.

**Table 8:** Length, bored position and external diameter of dead internode sections without evidence of bee nest processing (abandoned after boring).

Bamboo Species/Variety	n	Section Length (cm)	Bore Hole Position (cm from Bottom)	Bore Hole Position (% from Bottom)	Diameter at the Middle (mm)
*Bambusa multiplex* f. *alphonso-karri*	0	-	-	-	-
*B. multiplex* f. *variegata*	1	36	18	50	21
*B. multiplex* var. *gracillima*	0	-	-	-	-
*B. multiplex*	0	-	-	-	-
*Phyllostachys pubescens* var. *heterocycla*	0	-	-	-	-
*Phyllostachys* sp. ^1^	1	24	8	33.3	26
*P. nigra* f. *punctata*	2	13, 20	1, 1	7.7, 5.0	26, 24
*P. nigra* var. *nigra*	0	-	-	-	-
*P. nigra* f. *boryana*	2	18, 23	10, 1	55.6, 4.3	26, 11
*P. pubescens*	1	25	3	12	22
*Semiarundinaria* sp. ^2^	0	-	-	-	-
*S. fastuosa* var. *fastuosa*	0	-	-	-	-
*S. yashadake*	2	41, 35	1, 1	2.4, 2.9	18, 17
*Tetragonocalamus quadrangularis*	0	-	-	-	-
Total	9	26.1 ± 9.4 (13–41)	4.9 ± 6.0 (1–18)	19.2 ± 21.3 (2.4–55.6)	21.2 ± 5.1 (11–26)

^1^ The signboard displayed the Japanese and scientific names “*Hime-hachiku*” and *Phyllostachys humilis* Muroi, respectively. ^2^ The signboard displayed the Japanese and scientific names “*Kenashi-narihira*” and *Semiarundinaria fastuosa* Makino, respectively.

**Table 9:** Length, bored position and external diameter of live internode sections without evidence of nest processing (abandoned after boring).

Bamboo Species/Variety	n	Section Length (cm)	Bore Hole Position (cm from Bottom)	Bore Hole Position (% from Bottom)	Diameter at the Middle (mm)
*Bambusa multiplex* f. *alphonso-karri*	9	30.9 ± 2.1 (27–35)	18.4 ± 4.4 (12–26)	60.2 ± 16.7 (38.7–96.3)	21.3 ± 1.6 (19–23)
*B. multiplex* f. *variegata*	0	-	-	-	-
*B. multiplex* var. *gracillima*	0	-	-	-	-
*B. multiplex*	0	-	-	-	-
*Phyllostachys pubescens* var. *heterocycla*	0	-	-	-	-
*Phyllostachys* sp. ^1^	5	28.2 ± 3.7 (24–33)	8.0 ± 4.9 (1–14)	29.5 ± 19.5 (3.3–56.0)	22.8 ± 1.9 (20–25)
*P. nigra* f. *punctata*	0	-	-	-	-
*P. nigra* var. *nigra*	0	-	-	-	-
*P. nigra* f. *boryana*	0	-	-	-	-
*P. pubescens*	1	31	15	48.4	22
*Semiarundinaria* sp. ^2^	3	22, 30, 26	1, 1, 1	4.5, 3.3, 3.8	23, 24 24
*S. fastuosa* var. *fastuosa*	1	25	1	4.0	23
*S. yashadake*	2	26, 37	1, 1	3.8, 2.7	23, 22
*Tetragonocalamus quadrangularis*	0	-	-	-	-
Total	21	29.3 ± 3.7 (22–37)	10.8 ± 8.4 (1–26)	36.2 ± 28.0 (2.7–96.3)	22.2 ± 1.6 (19–25)

The values are in the form mean ± SD (range) for the materials with n = 5 or more, and actual values are given for the materials with n = 4 or less. ^1^ The signboard displayed the Japanese and scientific names “*Hime-hachiku*” and *Phyllostachys humilis* Muroi, respectively. ^2^ The signboard displayed the Japanese and scientific names “*Kenashi-narihira*” and *Semiarundinaria fastuosa* Makino, respectively.

**Table 10:** Arthropods secondarily utilizing internode sections without bee nest processing.

Bamboo Species/Variety	*A. gibbifrons*	Lepdopteran Larvae	Lepidopteran Larvae + Spiders	Shield Bug
*Bambusa multiplex* f. *alphonso-karri*	-	5	-	-
*Phyllostachys* sp. ^1^	-	5	-	-
*P. nigra* f. *punctata*	-	2	-	-
*P. nigra* f. *boryana*	1	-	-	-
*P*. *pubescens*^2^	-	1	-	-
*Semiarundinaria* sp. ^3^	-	-	-	1
*S*. *fastuosa* var. *fastuosa* ^1^	-	-	1	-

^1^ The signboard displayed the Japanese and scientific names “*Hime-hachiku*” and *Phyllostachys humilis* Muroi, respectively. ^2^ Fresh culm. ^3^ The signboard displayed the Japanese and scientific names “*Kenashi-narihira*” and *Semiarundinaria fastuosa* Makino, respectively.

## Data Availability

All original row data are available in the present study and Supplementary Material S1.

## Supplementary Materials

The following supporting information can be downloaded at https://www.mdpi.com/article/10.3390/insects16080807/s1, Supplementary Material S1: Row data for site 1.
